# Supplementary material for: Characterization of Natural Anthocyanin Indicator Based on Cellulose Bio-Composite Film for Monitoring the Freshness of Chicken Tenderloin
Source: Molecules. 2022 Apr 25;27(9):2752. doi: 10.3390/molecules27092752 (PMC9103511; doi:10.3390/molecules27092752)
Supplement: Supplementary file 1 [file molecules-27-02752-s001.zip › molecules-1698903-supplementary.pdf]

Supplementary materials

Characterization of Natural Anthocyanin Indicator Based on Cellulose Bio-Composite Film for Monitoring the Freshness of Chicken Tenderloin

Athip Boonsiriwit [1,2], Pontree Itkor [1], Chanutwat Sirieawphikul [2] and Youn Suk Lee [1,*]

Table S1. The overview of type of biopolymer for anthocyanin bio-composite film fabrication

Biopolymer	Source of anthocyanins	Anthocyanins content (w/w)	Purpose	Reference
Agarose	Jamun peel	NM	pH senescing indicator	**Talukder**, ***et al.*** **[1]**
Alginate	Black chokeberry	8,16,28 and 40%	Radical scavenging film	**Kim**, ***et al.*** **[2]**
Arrowroot starch	Blackberry pulp	20, 30 and 40%	Anti-oxidation film	**Nogueira**, ***et al.*** **[3]**
Bacterial cellulose	*Echium amoenum* flowers	no mentioned	pH senescing indicator	**Mohammadalinejhad**, ***et al.*** **[4]**
κ-Carrageenan/locust bean gum	Cranberry	20%	Anti-oxidation film	**Zepon**, ***et al.*** **[5]**
Cassava starch	Bayberry	1, 2, 3 and 4%	pH senescing indicator	**Yun**, ***et al.*** **[6]**
Cellulose/chitosan	Black carrot	1.05%	pH senescing indicator	**Ebrahimi Tirtashi**, ***et al.*** **[7]**
Chitosan/PVA	Black carrot	1%	Anti-microbial film	**Koosha and Hamedi [8]**
Corn starch	Black bean seed and red cabbage	0.05%	pH senescing indicator	**Prietto**, ***et al.*** **[9]**
Furcellaran	Blueberry and elderberry	5 - 20%	pH senescing indicator	**Jamróz**, ***et al.*** **[10]**
Gelatin	Black rice bran	0.5, 1, 2 and 2.5 %	Anti-oxidation film and pH senescing indicator	**Ge**, ***et al.*** **[11]**

Gelatin	Blood orange peel	5 and 10%	Anti-oxidation film	**Jridi, *et al.* [12]**
Guar gum	Grape pomace	0.5, 1, 2.5, 5 and 7.5 %	Anti-oxidation and anti-microbial film	**Saurabh, *et al.* [13]**
Konjac glucomannan/chitosan	Mulberry	3, 6 and 9%	Anti-oxidation and anti-microbial film	**Sun, *et al.* [14]**

Reference

1. Talukder, S.; Mendiratta, S.; Kumar, R.; Agrawal, R.; Soni, A.; Luke, A.; Chand, S. Jamun fruit (Syzgium cumini) skin extract based indicator for monitoring chicken patties quality during storage. *Journal of Food Science and Technology* **2020**, *57*, 537-548.
2. Kim, S.; Baek, S.-K.; Song, K.B. Physical and antioxidant properties of alginate films prepared from Sargassum fulvellum with black chokeberry extract. *Food Packaging and Shelf Life* **2018**, *18*, 157-163.
3. Nogueira, G.F.; Fakhouri, F.M.; Velasco, J.I.; de Oliveira, R.A. Active edible films based on arrowroot starch with microparticles of blackberry pulp obtained by freeze-drying for food packaging. *Polymers* **2019**, *11*, 1382.
4. Mohammadalinejhad, S.; Almasi, H.; Moradi, M. Immobilization of Echium amoenum anthocyanins into bacterial cellulose film: A novel colorimetric pH indicator for freshness/spoilage monitoring of shrimp. *Food Control,* **2020**, *113*, 107169, doi:https://doi.org/10.1016/j.foodcont.2020.107169.
5. Zepon, K.M.; Martins, M.M.; Marques, M.S.; Heckler, J.M.; Morisso, F.D.P.; Moreira, M.G.; Ziulkoski, A.L.; Kanis, L.A. Smart wound dressing based on κ–carrageenan/locust bean gum/cranberry extract for monitoring bacterial infections. *Carbohydrate polymers* **2019**, *206*, 362-370.
6. Yun, D.; Cai, H.; Liu, Y.; Xiao, L.; Song, J.; Liu, J. Development of active and intelligent films based on cassava starch and Chinese bayberry (Myrica rubra Sieb. et Zucc.) anthocyanins. *RSC advances* **2019**, *9*, 30905-30916.
7. Ebrahimi Tirtashi, F.; Moradi, M.; Tajik, H.; Forough, M.; Ezati, P.; Kuswandi, B. Cellulose/chitosan pH-responsive indicator incorporated with carrot anthocyanins for intelligent food packaging. *International Journal of Biological Macromolecules* **2019**, *136*, 920-926, doi:https://doi.org/10.1016/j.ijbiomac.2019.06.148.
8. Koosha, M.; Hamedi, S. Intelligent Chitosan/PVA nanocomposite films containing black carrot anthocyanin and bentonite nanoclays with improved mechanical, thermal and antibacterial properties. *Progress in Organic Coatings,* **2019**, *127*, 338-347.
9. Prietto, L.; Mirapalhete, T.C.; Pinto, V.Z.; Hoffmann, J.F.; Vanier, N.L.; Lim, L.-T.; Dias, A.R.G.; da Rosa Zavareze, E. pH-sensitive films containing anthocyanins extracted from black bean seed coat and red cabbage. *Lwt* **2017**, *80*, 492-500.
10. Jamróz, E.; Kulawik, P.; Guzik, P.; Duda, I. The verification of intelligent properties of furcellaran films with plant extracts on the stored fresh Atlantic mackerel during storage at 2 °C. *Food Hydrocolloids,* **2019**, *97*, 105211, doi:https://doi.org/10.1016/j.foodhyd.2019.105211.
11. Ge, W.; Zhao, Y.; Kong, X.; Sun, H.; Luo, M.; Yao, M.; Wei, B.; Ji, S. Combining salicylic acid and trisodium phosphate alleviates chilling injury in bell pepper (Capsicum annuum L.) through enhancing fatty-acid desaturation efficiency and water retention. *Food Chemistry* **2020**, *327*, 127057, doi:https://doi.org/10.1016/j.foodchem.2020.127057.

12. Jridi, M.; Boughriba, S.; Abdelhedi, O.; Nciri, H.; Nasri, R.; Kchaou, H.; Kaya, M.; Sebai, H.; Zouari, N.; Nasri, M. Investigation of physicochemical and antioxidant properties of gelatin edible film mixed with blood orange (*Citrus sinensis*) peel extract. *Food Packaging and Shelf Life,* **2019**, *21*, 100342, doi:https://doi.org/10.1016/j.fpsl.2019.100342.
13. Saurabh, C.K.; Gupta, S.; Variyar, P.S. Development of guar gum based active packaging films using grape pomace. *Journal of food science and technology* **2018**, *55*, 1982-1992.
14. Sun, J.; Jiang, H.; Wu, H.; Tong, C.; Pang, J.; Wu, C. Multifunctional bionanocomposite films based on konjac glucomannan/chitosan with nano-ZnO and mulberry anthocyanin extract for active food packaging. *Food Hydrocolloids* **2020**, *107*, 105942.
